# Protonation-Independent Charge Transport Across Diphenylamine Single-Molecule Junctions

**DOI:** 10.1021/acs.jpclett.4c03299

**Published:** 2025-01-27

**Authors:** Yaran Cheng, Jiahao Wang, Yangyang Shen, Haixing Li

**Affiliations:** † Department of Physics, City University of Hong Kong, Kowloon 999077, Hong Kong, China; ‡ Frontier Institute of Science and Technology, Xi’an Jiaotong University, Yanxiang Road 99, Xi’an 710045, China

## Abstract

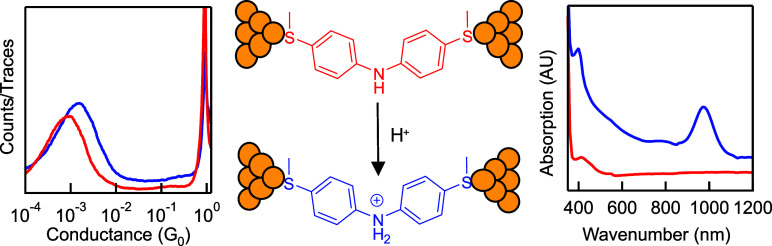

Amines are one of the most ubiquitous functional groups in molecular junctions; however, the exact regulation of the charge transport through the protonation state of an amine group in the junction backbone remains elusive. We address this question here by designing a diphenylamine molecular backbone and experimentally investigating how protonation of the central amine group affects the charge transport. Our ultraviolet–visible spectroscopy measurements demonstrate the protonation reaction of the diphenylamine compound in the presence of either trifluoroacetic acid or HCl, and we observe a consistent trend of a modestly increased conductance for diphenylamine in the presence of acid, indicating that a protonated amine group in a diphenylamine backbone slightly enhances the electron conduction. We further investigate the charge transport across diphenylamine under a series of applied tip bias voltages between −0.9 to 0.9 V in an electrochemical environment in the absence and presence of acid for determining the frontier molecular orbital alignment with the Fermi level and the coupling coefficient between the molecule and the electrodes. Our finding shows that the highest occupied molecular orbital (HOMO) is the dominating transport channel of the diphenylamine junction, and a modest conductance increase is an outcome of the HOMO resonance energy moving closer to the Fermi level upon protonation of the amine.

Regulation of charge transport across single-molecule wires is of both fundamental importance for understanding the electron conduction at nanoscale and practical relevance for applying organic materials in constructing electronic devices.^1,2^ Various factors impact the charge transport, such as material of the electrodes, light illumination,^3−5^ mechanical force,^6^ a high bias voltage applied,^7−9^ pH of the solution,^10−12^ and the choice of the anchor group,^13,14^ as a change in the molecule/metal interaction, as well as in the redox status, protonation state, or conformation of the molecule, can all affect the charge transport processes across molecular junctions. As it is common that more than one of such change occurs simultaneously in a system under a stimulus, dissecting the roles of each element in contributing to the overall change in the charge transport becomes difficult. For example, previous studies have shown that the conductance of molecular junctions containing primary amine and carboxylic acid groups as side groups and secondary amine in the backbone was influenced by the pH of the solution.^15,16^ Due to the protonation reactions at multiple sites (primary and secondary amines and carboxylic acids) of such backbones, we cannot directly make conclusions about the role of one specific protonated chemical group in controlling the charge transport. Such knowledge is valuable for designing new electronic materials; we are therefore interested in designing simple structures where we focus on the protonation reaction of one specific unit and how its protonation state affects the overall single-molecule conductance of the molecular junction.

Schlicke and Herrmann et al. reported first-principles calculations of the transmission of neutral as well as protonated thiol-terminated diphenylamines attached to Au electrodes.^17^ The authors showed that when the bridging amine group becomes protonated, the transmission between the highest occupied molecular orbital (HOMO) and the lowest unoccupied molecular orbital (LUMO) energies is reduced by more than 1 order of magnitude and the HOMO–LUMO gap is increased by 0.4 eV. Besides, the authors calculated the local current contributions and indicated that the nitrogen atom in the center of the backbone participates in electron transport when amine is neutral and participates significantly less when amine becomes protonated. The authors further showed a different dihedral angle between the two phenyl rings for the neutral and the protonated molecules: ∼40–65° versus ∼90°. The two phenyl planes nearly perpendicular to each other in the protonated species suggests a substantially suppressed **π** conjugation across the molecule.^18^ Taken together, these calculation results suggested a lower single-molecule junction conductance for diphenylamine when the molecule becomes protonated. Based on Schlicke and Herrmann’s computational study,^17^ we are motivated to experimentally study the effect of protonation on the single-molecule conductance of a diphenylamine junction; for clean rupture of gold atomic contacts and well-defined formation and rupture of single-molecule junctions in scanning tunneling microscope-based break junction (STM-BJ) measurements,^19−21^ we replace the thiol with thiomethyl anchor groups in this work.

In a separate study, Li et al. explored oligo[n]emeraldine wires of different lengths (n = 2–7).^22^ The authors demonstrated that by performing STM-BJ measurements in propylene carbonate (PC) under a positive tip bias of 500 mV with added trifluoroacetic acid (TFA), oligo[n]emeraldine molecules become oxidized to exhibit radical amine cations. The authors further showed that such oxidized wires exhibit increasing conductance with increasing length, a behavior the authors ascribed to one-dimensional topological insulators. As the molecule bearing a single functional unit was not discussed by Li et al., possibly because no pair of radicals can be generated in that case, we herein study the charge transport properties of a diphenylamine unit, the n = 1 case for the oligo[n]emeraldine.

In this work, we apply the STM-BJ technique for measuring the single-molecule conductance of neutral and protonated diphenylamine junction. We apply two different acids, TFA and HCl, for protonating the diphenylamine molecule and observe the appearance of new absorption peaks in UV–vis spectra in both cases, indicating the protonation reaction at the amine group. In conductance measurements, we see a slight increase by less than a factor of 2 for the single-molecule conductance of the diphenylamine junction in the presence of acid, suggesting that the protonated amine likely does not suppress but instead enhances the charge transport. We further experimentally probe the coupling coefficient between the molecule and the electrode as well as the energy alignment between the frontier molecular orbital and the Fermi level. We find that upon protonation, the coupling coefficient remains similar (∼15% decrease), and the slight increase in conductance mainly results from the frontier orbital HOMO moving closer to the Fermi level by ∼0.59 eV. By evaluating the charge transport properties of diphenylamine in two different acids, we provide new perspectives on the widely used amine functional group, which may motivate new studies and applications of amines as active electronic components.

We first measure the conductance of **1** in solvent 1,2,4-trichlorobenzene (TCB) using the scanning tunneling microscope-based break junction (STM-BJ) technique.^18,23,24^ The single-molecule junction of **1**, a diphenylamine backbone terminated by thiomethyl groups for robust binding to the Au electrodes, is illustrated in Figure 1a. We observe plateaus at ∼10^–3^ G_0_ with an ∼0.4 nm elongation length in individual conductance-displacement traces and a two-dimensional (2D) conductance histogram of **1** (Figure 1b and 1d). In the compiled one-dimensional (1D) conductance histograms for **1** measured in TCB under 90 mV, 450 mV, and 900 mV tip bias voltages, we see a slight increase in peak conductance value with increasing tip bias voltage (Figure 1c; 2D conductance histograms are provided in Figures 1d and S1), agreeing with the coherent electron tunneling process described by the Landauer model.^25^ Such a phenomenon has been observed in previous single-molecule conductance studies,^26,27^ and several molecules have also been reported to exhibit a constant conductance under varied bias voltages between 0.2 and 1.4 V.^11,27^ We note that, from cyclic voltammetry measurements of **1**, we observe two oxidation and two reduction events (Figure S2), in agreement with previous reports of similar compounds.^22^ Chen et al. has suggested that a backbone containing three secondary amine groups was oxidized in STM-based conductance measurements.^28^ In a separate study, the authors showed that in conductance measurements of oligo[n]emeraldine (n = 2–7) in an ionic solution with added TFA under a positive 500 mV tip bias, the molecules become oxidized.^22^ However, we emphasize that under the different voltages that we have applied in measurements performed in the presence or absence of acid in the current work, **1** is likely not oxidized. This is consistent with our observation that no abrupt change in the charge transport properties of **1** is seen under varying bias voltages. This also agrees with the previous reports, as pairs of radicals are formed when oligo[n]emeraldine (n = 2–7) becomes oxidized; however, in our experiment, the diphenylamine (n = 1 case for oligo[n]emeraldine) structure contains only one secondary amine and thus the same form of radical pair(s) cannot be generated.^29^ Given this observation of an increase by a factor of 1.7 in conductance measured under 900 mV compared to that measured under 90 mV, we also carried out conductance measurements of **1** in the presence of acid under both low and high bias voltages.

**Figure 1:**
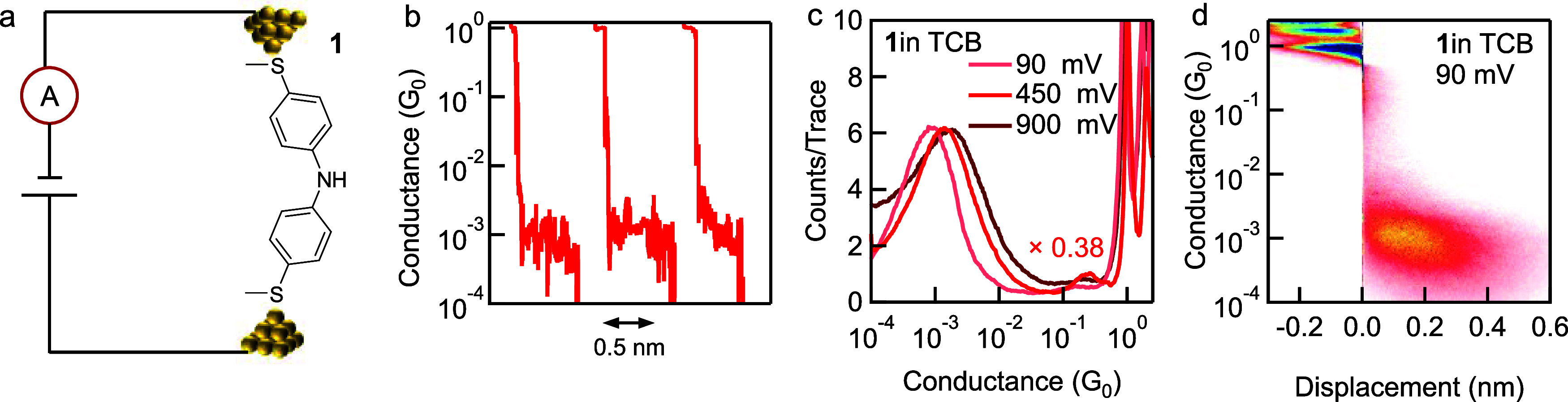
(a) Schematic of STM-BJ measurements of molecule **1**. (b) Typical individual traces from a single-molecule conductance measurement of **1** in TCB. (c) 1D conductance histograms of **1** measured in TCB under 90 mV, 450 mV, and 900 mV tip biases. (d) 2D conductance histogram of **1** measured in TCB under 90 mV tip bias.

To evaluate the protonation reaction of **1**, we compare the ultraviolet–visible (UV–vis) absorption spectra of solutions of **1** in the absence and presence of an acid. We use two acids, TFA and HCl, in this work. When TFA is added into the solution of **1** to achieve a molar ratio between **1** and TFA of 1:300 in TCB solvent, a new peak appears at ∼970 nm (Figure 2a). When the concentration of TFA is increased further, the solution becomes opaque, and the result of UV–vis is no longer accurate. Therefore, we do not analyze UV–vis results of **1** obtained under molar ratio higher than 300 between TFA and **1**. Next, when HCl is added into the solution of **1** in ethanol (EtOH), we observe two new peaks at ∼760 nm and ∼950 nm in the UV–vis spectra and the intensity of these two peaks increases with increasing concentration of HCl (Figure 2b). These results show that **1** is likely protonated under such concentrations of either TFA or HCl (the structure of protonated **1** is given in Figure 2c and is labeled as prot. **1**), and the new absorption peaks at larger wavelength indicate a reduction in the optical gap for protonated **1** than that of **1**. For completeness, we also introduce a base into the solution of **1** for evaluating any possible impact of a basic environment on **1**, such as the possibility of the formation of deprotonated **1**. We do not see any changes in the UV–vis spectra of **1** with added base, in the case of either triethylamine (TEA) added in TCB or NaOH added in EtOH (Figure S3(c–d)).

**Figure 2:**
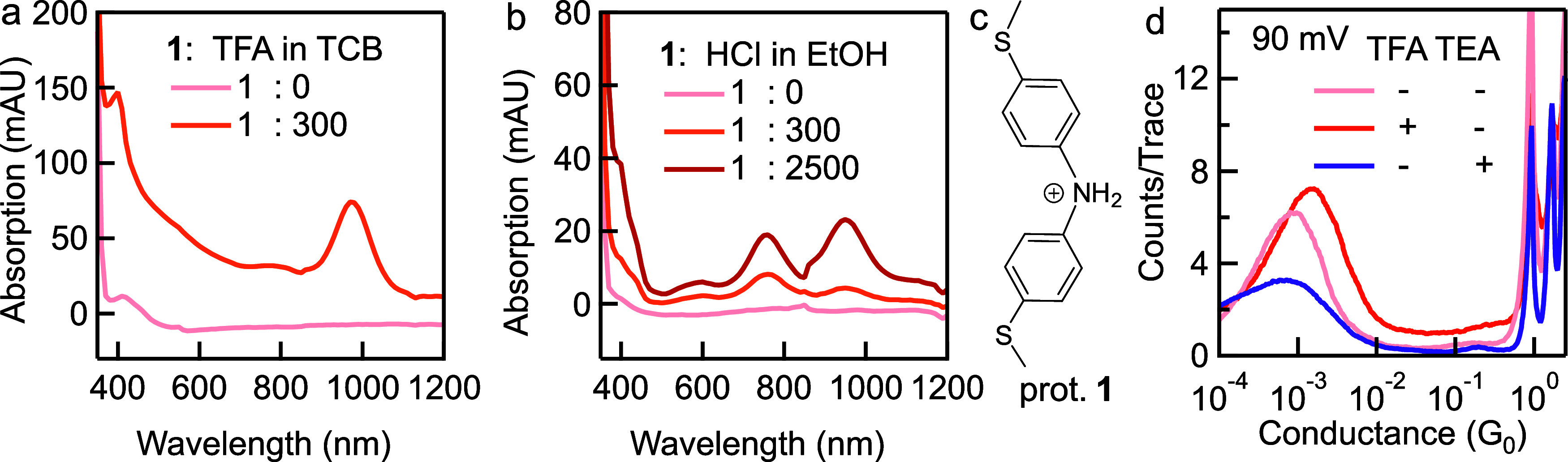
UV–vis spectra for 0.1 mM **1** measured under a molar ratio (a) between **1** and TFA of 1:0 and 1:300 in TCB, and (b) between **1** and HCl of 1:0, 1:300, and 1:2500 in EtOH. For clarity, the absorption data for 350–1200 nm is displayed here; complete spectra for 250–1200 nm are provided in Figure S3(a–b). (c) Chemical structure of protonated **1**. (d) 1D conductance histograms of **1** measured in TCB in the absence of acid or base (pink), in the presence of acid with a molar ratio between **1** and TFA of 1:2500 (orange), and in the presence of base with a molar ratio between **1** and TEA of 1:2500 (purple) under 90 mV.

We next carry out single-molecule conductance measurements of **1** in the presence of acid (TFA or HCl), and we additionally perform measurements of **1** in the presence of base (TEA or NaOH) for comparison. Specifically, we (i) add concentrated HCl aqueous solution or NaOH pellet to a solution of **1** in EtOH to achieve a molar ratio of 1:2000 between **1** and acid/base, or (ii) add organic acid TFA or organic base TEA into a solution of **1** in TCB to achieve a molar ratio of 1:2500 between **1** and acid/base. We show a comparison of three conductance histograms for **1** measured under 90 mV in the absence of acid or base, in the presence of acid TFA, and in the presence of base TEA in Figure 2d; we summarize the conductance values determined from measurements performed under a low or high bias voltage in a basic, neutral, or acidic environment in Table 1. The related 1D and 2D conductance histograms are given in Figures S4–S6. We notice that a modest change of less than a factor of 2 in conductance values of **1** is seen in all measurements performed in basic, neutral, or acidic environments under either low or high bias voltage. Specifically, when we compare the results obtained in the absence and presence of base, we do not observe a systematic trend. For measurements performed in the absence and presence of acid, conductance shows a consistent trend under either a low 90 mV or a high 900 mV bias voltage: we see no change or a slight increase to a factor of up to 2 in the conductance of **1** when acid is added (Table 1). Our results indicate that addition of acid or base in the molecular solution cannot effectively regulate the charge transport through a diphenylamine junction.

**Table 1:** Measured Single-Molecule Junction Conductance Values of **1**

Solvent, molar ratio between **1** and added acid or base	Conductance (×10^–3^ G_0_) under 90 mV	Conductance (×10^–3^ G_0_) under 900 mV
TCB, **1**:TEA = 1:2500	0.6	1.3
TCB	1	1.7
TCB, **1**:TFA = 1:2500	1.4	1.7
		
EtOH, **1**:NaOH = 1:2000	0.4	1.2
EtOH	1	1
EtOH, **1**:HCl = 1:2000	1	2

Previous works have shown that when the STM-BJ measurements were performed in an ionic environment with a wax-coated tip, conductance of a molecular junction is dependent on both the magnitude and polarity of the applied bias.^30^ In the work of Capozzi et al.,^31^ the authors show that when a dense electric double layer is formed at the solution/tip interface, a bias polarity-dependent shift in the resonance energy of the molecular junction occurs, enabling a determination of the transmission function in an energy range near the Fermi level. We follow the method established by Capozzi et al. to extract the transmission values from the STM-BJ experiments, assuming a coherent tunneling process through the single-molecule junction:
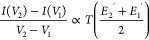
where *T*(*E*) is the energy-dependent transmission function, *V* is the bias voltage, and *I* is the current. We perform conductance measurements of **1** in propylene carbonate (PC) with a wax-coated tip at tip bias in intervals of 90 mV ranging from −900 to 900 mV (1D and 2D histograms are provided in Figures S7–S9). The STM Au tip is insulated with Apiezon wax with an ∼1 μm^2^ area of the tip apex exposed for eliminating the background ionic current. We determine the peak conductance value from Gaussian fits to the 1D conductance histograms and plot the conductance of **1** versus the applied tip voltage in Figure 3a. We see a slight increase in conductance with increasing positive tip biases and an almost unchanged conductance under negative tip biases, suggesting that charge transport through **1** is HOMO-dominated.^22^ In the inset of Figure 3a, we plot the transmission values extracted from the conductance measurements against the energy. We assume that the charge transport is dominated by a single level and fit this data to a single Lorentzian function,
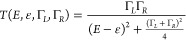
where ε is the energy of the frontier molecular orbital relative to the Fermi level, and Γ_R_ (Γ_L_) is the coupling coefficient to the right (left) electrode. We approximate that Γ_R_ = Γ_L_ and Γ_R_ (Γ_L_) is independent of energy. Then from the single Lorentzian fit, we obtain HOMO resonance ∼ −1.84 eV for **1**. This result agrees with previous findings that show a HOMO-dominated charge transport for thiomethyl-terminated π-conjugated molecules.^32,33^

**Figure 3:**
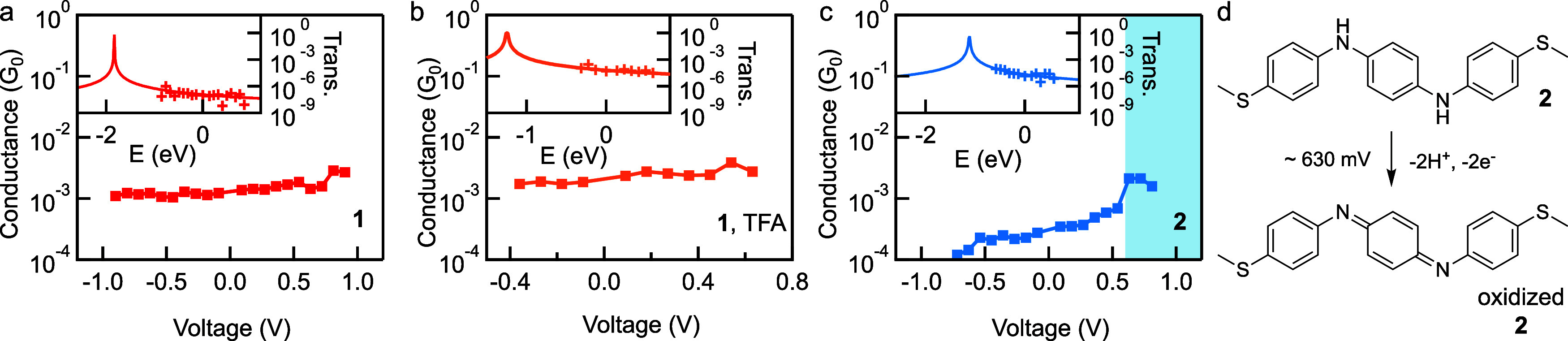
Main panels: peak conductance value versus applied voltage for (a) **1**, (b) **1** with added TFA at a molar ratio of 1: TFA = 1:2500, and (c) **2**. The peak conductance values are determined from measurements conducted with a wax-coated tip in PC solvent. Insets: extracted transmission values from the conductance features for (a) **1**, (b) **1** with added TFA, and (c) **2** plotted against energy. Solid lines in the insets are fits to single Lorentzian function. The shaded area in (c) indicates that 2 is oxidized under voltage >540 mV, and the conductance values measured for the oxidized **2** were not included in the extraction of transmission values for **2**. (d) Chemical structures of **2** and oxidized **2**.

We next investigate whether the addition of acid will alter the conductance versus the bias voltage trend that we observed for **1**. We perform conductance measurements of **1** with addition of TFA at molar ratio between **1** and TFA of 1:2500 with a wax-coated tip in PC at tip bias in intervals of 90 mV ranging from −540 to 720 mV (1D and 2D histograms are provided in Figures S10–S12). We note that under high bias voltages, either positive above 630 mV or negative below −360 mV, we no longer form robust junctions as we do not see clear molecular signatures in conductance histograms; this phenomenon that the formation of stable molecular junctions is prevented when a high bias voltage is applied has been seen previously.^31,34^ Within the applied bias range of −360 mV to 630 mV, we again observe a slight increase in conductance with increasing tip bias voltage (Figure 3b). This result shows that the charge transport through protonated **1** is also HOMO-dominated with a HOMO resonance determined to be ∼−1.25 eV. Thus, we see a closer energy alignment between the Fermi level and the HOMO for protonated **1** in comparison to that determined for **1** in the absence of acid. Additionally, we observe a slight decrease in the coupling coefficient Γ for **1** when **1** becomes protonated. Experimentally determined ε and Γ are summarized in Table 2. Overall, these results suggest that the observed insubstantial increase in the conductance of **1** in an acidic environment likely results from a closer alignment between the frontier HOMO and the Fermi level.

**Table 2:** Coupling Coefficient Γ and Level Alignment ε for **1**, **1** in the Presence of Acid, and **2** Determined from STM-BJ Experiments Performed with a Wax-Coated Tip in PC

Molecule	ε (eV)	Γ (×10^–4^ eV)
**1**	–1.84	6.32
Prot. **1** (**1**:TFA = 1:2500)	–1.25	5.35
**2**	–1.12	1.66

In order to verify our energy alignment results obtained from the conductance measurements performed in an ionic environment, we further test a control molecule **2** (chemical structure is given in Figure 3d right). We perform the conductance measurements of **2** in PC under a tip bias in intervals of 90 mV from −900 to 810 mV (1D and 2D histograms are given in Figures S13–S15). We again see that the conductance increases with increasing tip bias voltage (Figure 3c), indicating a HOMO-dominated charge transport in **2**. **2** is oxidized under a bias voltage above ∼540 mV, as reported previously^22,28^ and indicated by the sharp increase in conductance in Figure 3c. Therefore, we exclude the conductance values obtained from measurements performed under a bias voltage of >540 mV when extracting the transmission values. We obtain the HOMO resonance for **2** ∼ −1.12 eV. A closer alignment of the Fermi level to the HOMO resonance for **2** than **1** agrees with the observation that the change in conductance of **2** is about 2 times the change in conductance of **1** across ∼1.5 V bias range. This position of HOMO resonance for **2** agrees with the previously published density functional theory (DFT)-based transmission calculations from Li et al.^22^ Our finding shows that the dominating transport channel HOMO is outside of our accessible applied bias voltage range, and we have not reached resonance transport in these measurements of **1** (in the absence or presence of acid) or **2**.

Our experiments reveal that the single-molecule conductance of diphenylamine junctions is only modestly regulated by the protonation reaction in the presence of acid, as the observed conductance increase upon protonation is less than a factor of 2. We further show that the dominant transport channel for a thiomethyl-terminated diphenylamine molecule is the HOMO, similar to other thiomethyl-terminated molecular junctions. When diphenylamine becomes protonated, the energy alignment between the HOMO and the Fermi level is reduced by ∼0.6 eV, while the coupling coefficient between the molecule and the electrodes is slightly decreased, thereby leading to an overall minor increase in conductance. Our results highlight that the protonation of one amine group in a molecular junction does not significantly alter the conductance of the junction, albeit the protonation induces a closer energy alignment between the frontier orbital and the Fermi level. The knowledge obtained in this work contributes to our fundamental understanding of the amine, a functional group that plays critical roles in biological macromolecules and synthetic compounds, and we envision that such understanding about the protonated amine and its electronic properties will be exploited in future design of electronic components.

## Supporting Information Available

Experimental procedures and additional figure (PDF)
