# Supplementary material for: Protonation-Independent Charge Transport Across Diphenylamine Single-Molecule Junctions
Source: J Phys Chem Lett. 2025 Jan 27;16(5):1247–52. doi: 10.1021/acs.jpclett.4c03299 (PMC11808790; doi:10.1021/acs.jpclett.4c03299)
Supplement: Supplementary file 1 — jz4c03299_si_001.pdf [file jz4c03299_si_001.pdf]

Supporting Information

Protonation-independent charge transport across diphenylamine single-molecule junctions

Yaran Cheng,[1] Jiahao Wang,[2] Yangyang Shen,*[2] Haixing Li,*[1]

[1]Department of Physics, City University of Hong Kong, Kowloon 999077, Hong Kong, China
[2]Frontier Institute of Science and Technology, Xi'an Jiaotong University, Yanxiang Road 99, Xi'an 710045, China

Table of Contents

I. Scanning Tunneling Microscope-based Break Junction Experiment Details 2
II. Additional Figures and Tables 3
III. References 14

I. Scanning Tunneling Microscope-based Break Junction Experiment Details

We applied a scanning tunneling microscope-based break junction (STM-BJ) technique for performing single-molecule conductance measurements at room temperature under ambient condition, as detailed in the supporting information of a previous publication.[1] For measurements performed in TCB or EtOH, a mechanically cut gold wire (Ø = 0.25 mm, 99.999%, Hebei Hongju Metal Materials Co. Ltd) was prepared as the gold tip. For measurements performed in PC, the gold tip was insulated with Apiezon wax (Model W, M&I Materials Limited, Manchester, UK). Details for making the wax-coated tips were described previously.[1] A steel specimen disc (Ø = 15 mm) was mechanically polished, followed by an evaporation of ~ 100 nm thick gold (99.999%, Hebei Hongju Metal Materials Co. Ltd) film on the top at a speed of ~ 1.0 Å/s with the use of a thermal evaporation system (Beijing Technol Science Co Ltd, Beijing, China). The steel disc was UV-ozone treated for 20 mins before each experiment. We collected conductance data at an acquisition rate of 40 kHz and a speed of 18-19 nm/s for withdrawing the tip.

Reagents propylene carbonate (PC; Aladdin, standard for GC, ≥ 99.7%), 1,2,4-trichlorobenzene (TCB; Aladdin, anhydrous, ≥ 99%, or Alfa Aesar, spectrophotometric grade, 99%), ethyl alcohol (EtOH; 95%, Anaqua), hydrochloric acid (HCl; Rcilabscan, 37%), sodium hydroxide (NaOH; Fuchen Chemistry Co. Ltd.), trifluoroacetic acid (TFA, Aladdin, standard for GC), and triethylamine (TEA, Aladdin, standard for GC, ≥ 99.5%) were used without any further purification. Molecule **1**[2] and **2**[3] were known compounds and synthesized according to the reported procedures. We performed the conductance measurements in solution when TCB or PC was used as the solvent, and we dropped the solution on the substrate and allowed the solvent to evaporate at room temperature prior to measurements when EtOH was used as the solvent. A solution of 0.1 mM of the target molecule in solvent TCB or PC was prepared prior to each measurement. For measurements of **1** performed in EtOH under acidic (basic) condition, we first prepared a solution of 0.1 mM **1** in EtOH, then we added an aqueous solution of 37% HCl (NaOH pellet) into the solution to reach a molar ratio between the molecule **1** and acid (base) of 1: 2000. For measurement of **1** performed in TCB under acidic (basic) condition, we first prepared a solution of 0.1 mM **1** in TCB, then we added TFA (TEA) into the solution to reach a molar ratio between the molecule **1** and acid (base) of 1: 2500. For each single-molecule conductance measurement, we collected 9,400-10,000 traces that were used for constructing 1D and 2D conductance histograms without data selection. For experiments performed in PC under varied tip bias voltages in the range of -900 mV to 900 mV, we collected 1,800-2,000 traces for each measured bias voltage.

II. Additional Figures and Tables

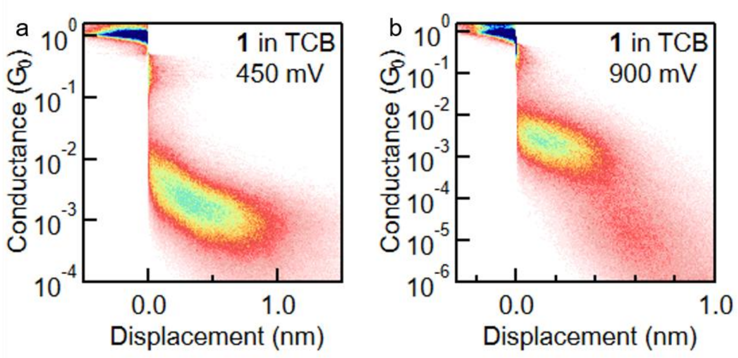

Figure S1. 2D conductance histograms of **1** measured in TCB under (a) 450 mV and (b) 900 mV tip bias voltages.

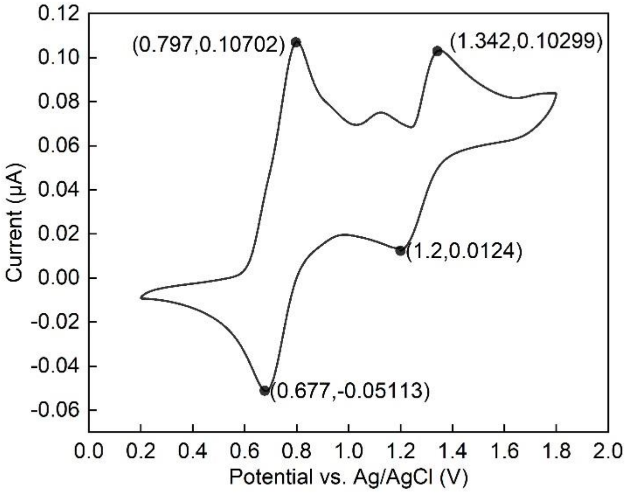

Figure S2. Cyclic voltammetry of **1** measured by using solutions of 5 mM **1** in acetonitrile with added NBu_4PF_6 in concentration of 0.1 M with glassy carbon disk electrode at a scan rate of 0.1 V/s. The counter-electrode was a platinum disk and the reference electrode was Ag/AgCl.

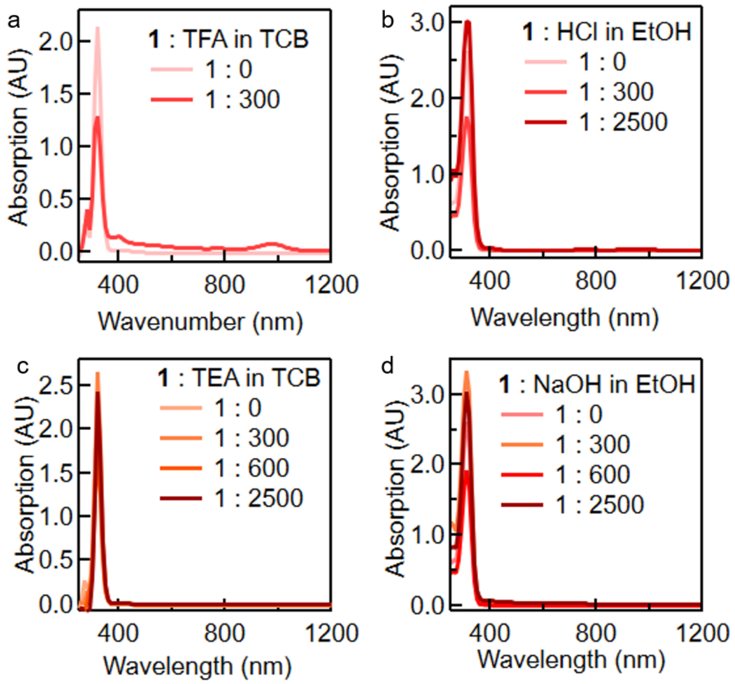

Figure S3. UV-vis spectra for 0.1 mM **1** measured under a molar ratio (a) between **1** and TFA of 1 : 0 and 1 : 300 in TCB, (b) between **1** and HCl of 1 : 0, 1 : 300, and 1 : 2500 in EtOH, (c) between **1** and TEA of 1 : 0, 1 : 300, 1 : 600, and 1 : 2500 in TCB, and (d) between **1** and NaOH of 1 : 0, 1 : 300, 1 : 600, and 1 : 2500 in EtOH. Magnification of the region between 350nm and 1200 nm for (a) and (b) are shown in Figure 2.

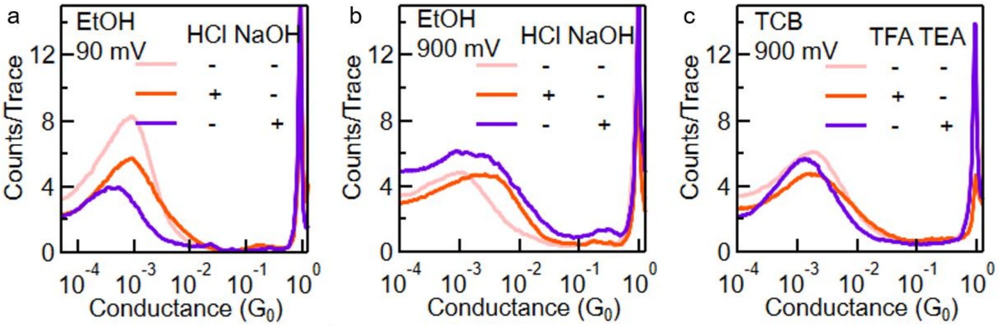

Figure S4. 1D conductance histograms of **1** measured in EtOH in the absence of acid or base, in the presence of HCl (molar ratio between **1** : HCl = 1 : 2000), and in the presence of NaOH (molar ratio between **1** : NaOH = 1 : 2000) under (a) 90 mV and (b) 900 mV bias voltages. (c) 1D conductance histograms of **1** measured in TCB in the absence of acid or base, in the presence of TFA (molar ratio between **1** : HCl = 1 : 2500), and in the presence of TEA (molar ratio between **1** : TEA = 1 : 2500) under 900 mV bias voltage.

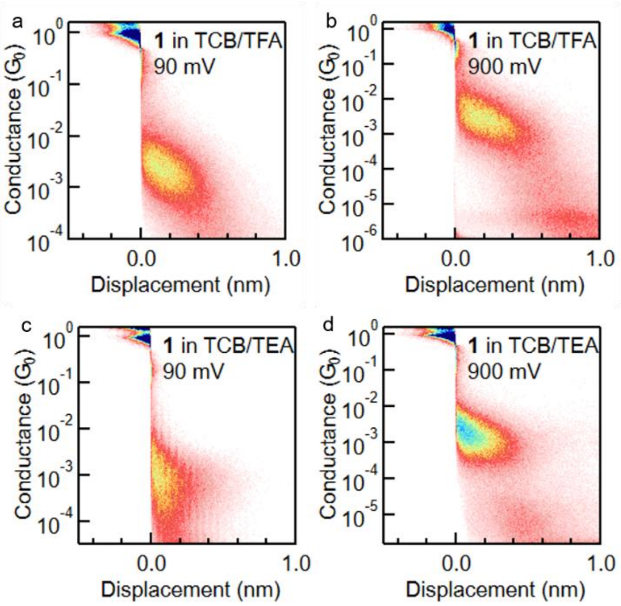

Figure S5. 2D conductance histograms of **1** in TCB with added TFA (a and b; molar ratio between **1** : TFA = 1 : 2500) or TEA (c and d; molar ratio between **1** : TEA = 1 : 2500) under 90 mV (left) and 900 mV (right) bias voltages.

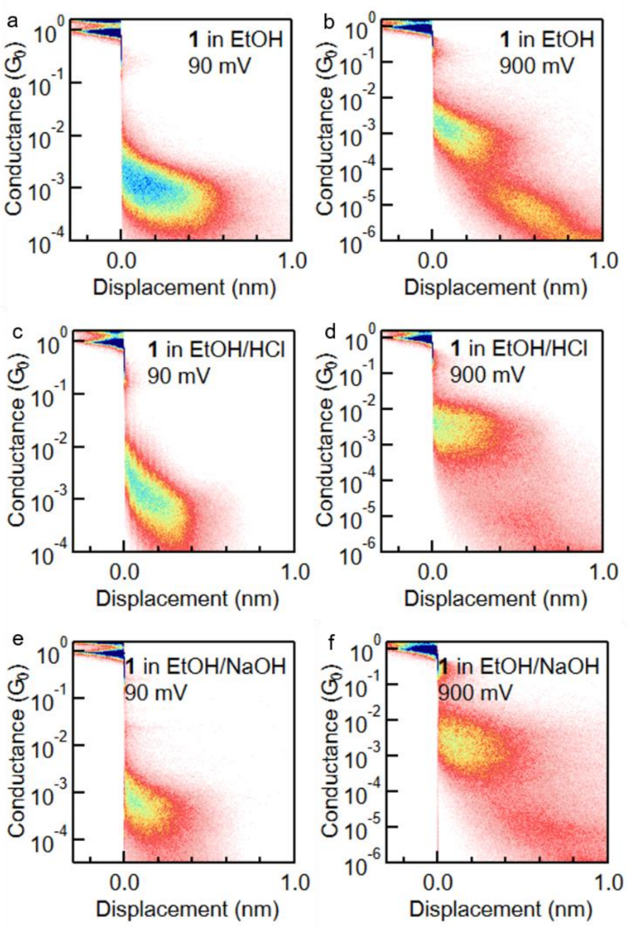

Figure S6. 2D conductance histograms of **1** in EtOH (a and b), in EtOH with added HCl (c and d; molar ratio between **1** : HCl = 1 : 2000), in EtOH with added NaOH (e and f; molar ratio between **1** : NaOH = 1 : 2000) under 90 mV (left) and 900 mV (right) bias voltages.

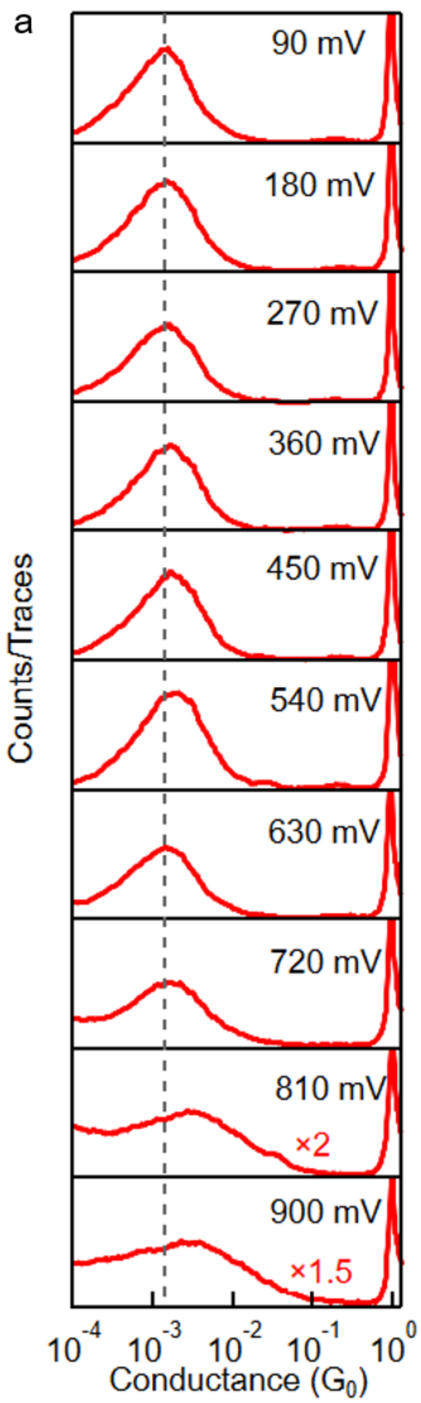

Figure S7. Logarithmically binned conductance histograms for molecule **1** measured in PC under (a) positive and (b) negative bias voltages.

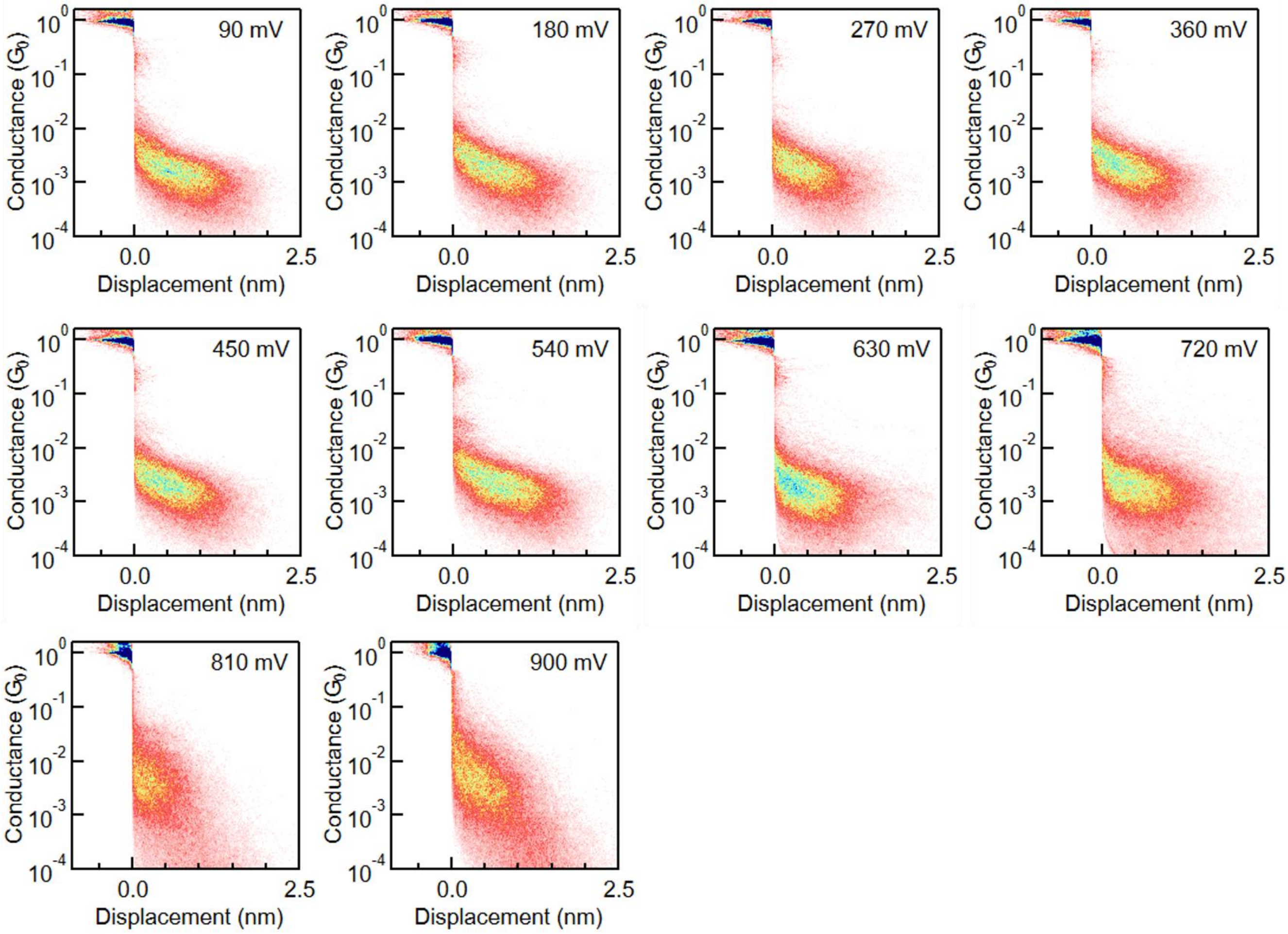

Figure S8. 2D conductance histograms of **1** measured in PC under positive tip bias voltages in the range of 90 – 900 mV.

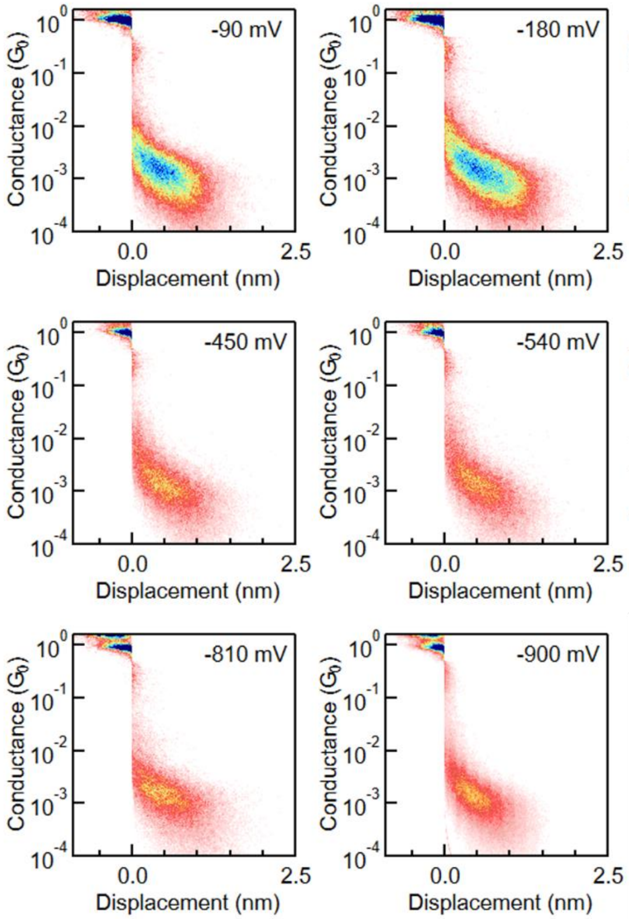

Figure S9. 2D conductance histograms of **1** measured in PC under negative tip bias voltages in the range from -90 mV to -900 mV.

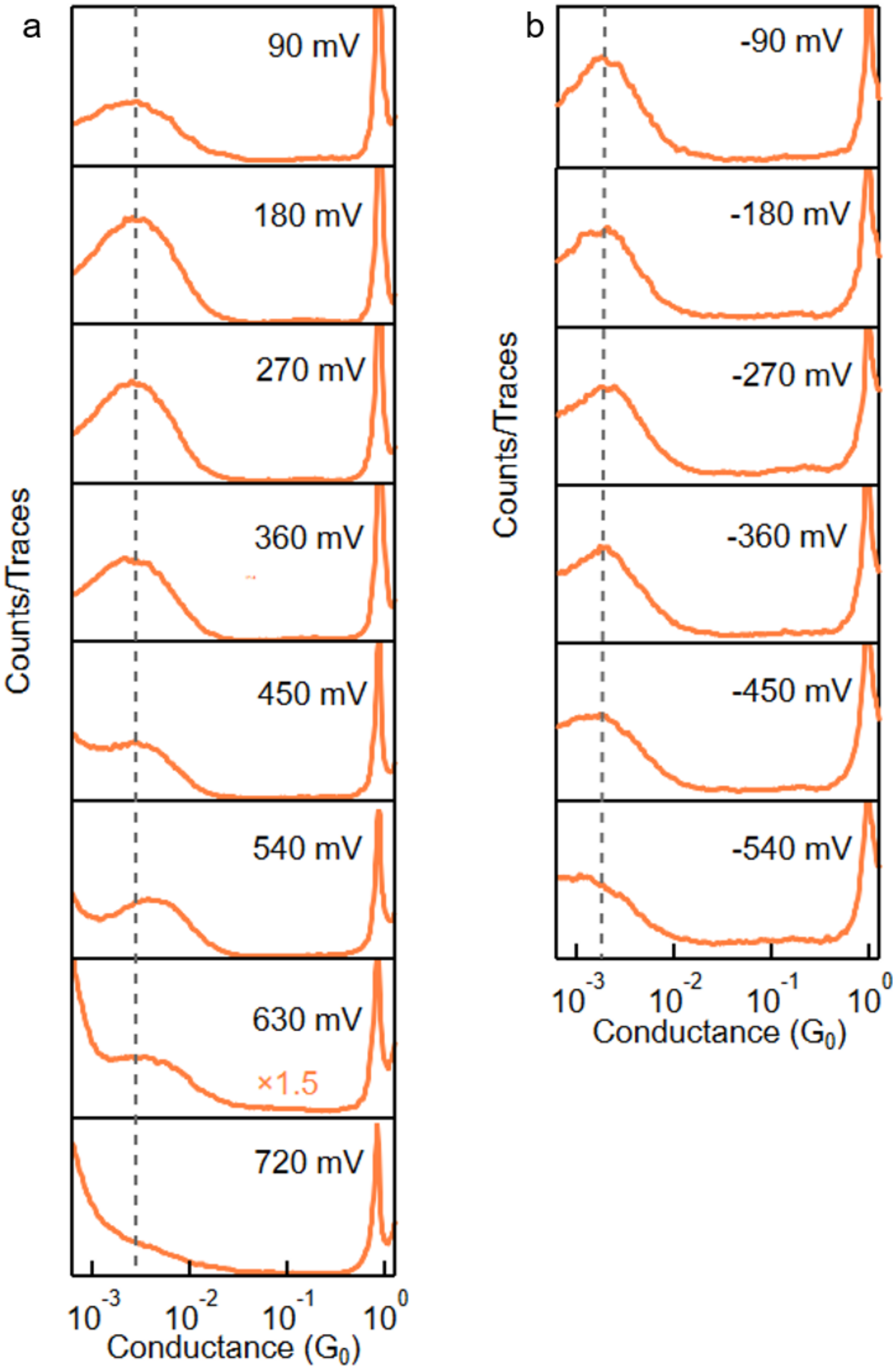

Figure S10. Logarithmically binned 1D conductance histograms for molecule **1** measured in PC with added TFA under a molar ratio between **1** and TFA of 1 : 2500 under (a) positive and (b) negative voltages. When the tip bias is increased to 720 mV or decreased to -540 mV, the molecular conductance peak is no longer visible.

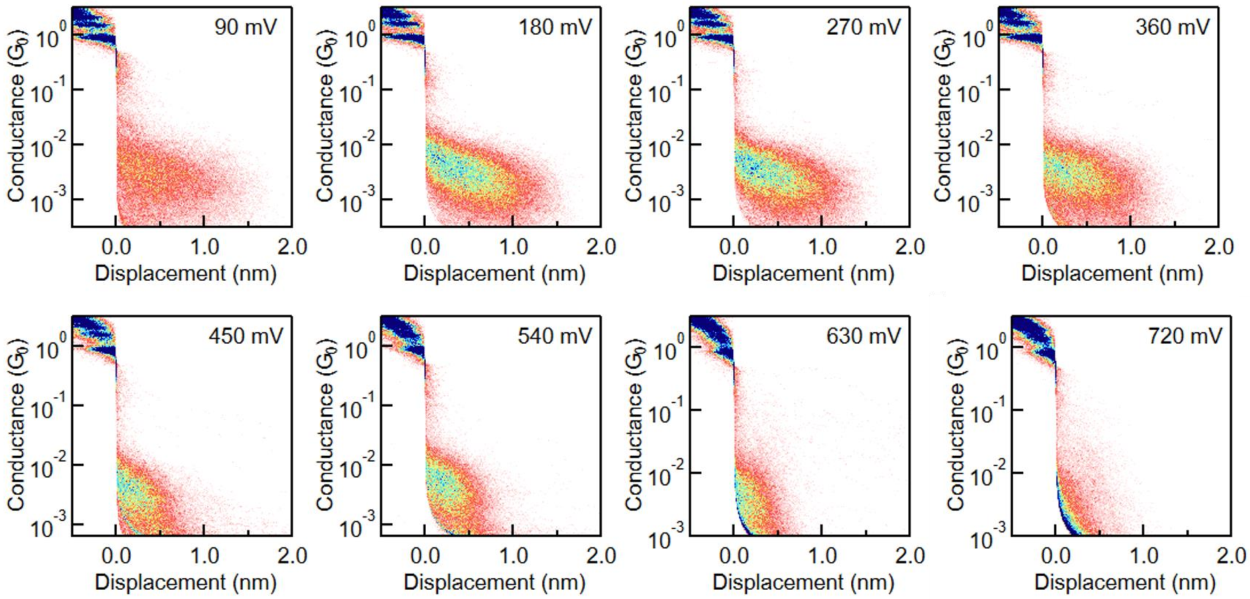

Figure S11. 2D conductance histograms of **1** measured in PC with added TFA under a molar ratio between **1** and TFA of 1 : 2500 under tip bias voltages in the range of 90 – 720 mV. We do not determine a single-molecule conductance value for measurements under 720 mV as no clear molecular junction formation is seen.

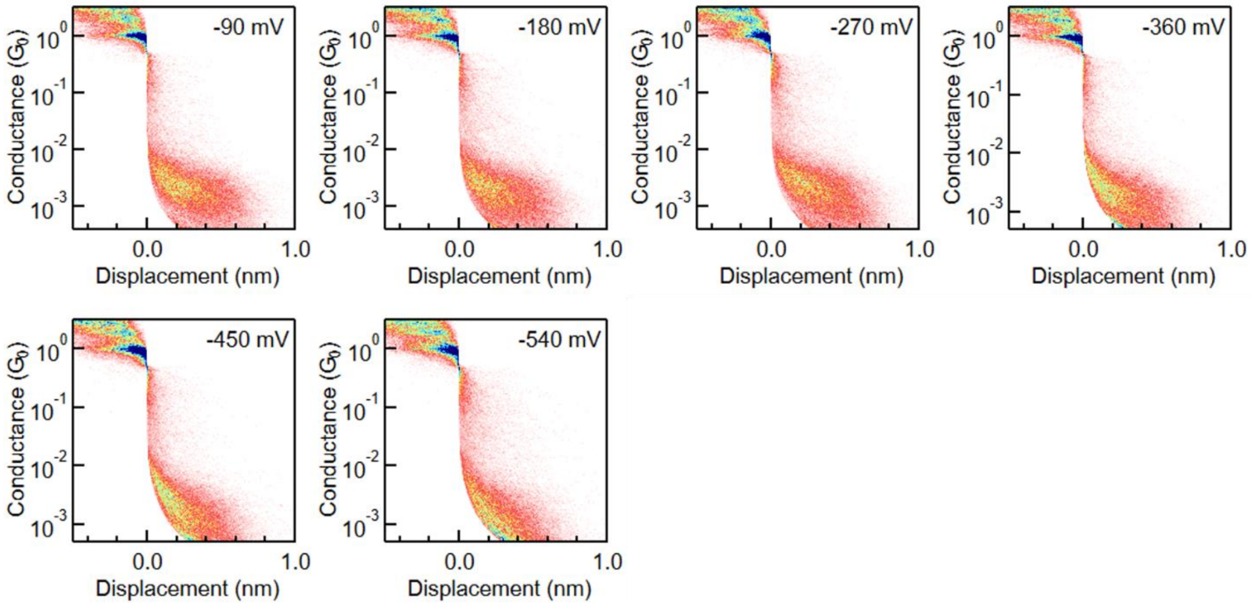

Figure S12. 2D conductance histograms of **1** measured in PC with added TFA under a molar ratio between **1** and TFA of 1 : 2500 under tip bias voltages in the range from -90 to -540 mV. We do not determine a single-molecule conductance value for measurements under -450 mV and -540 mV as no clear molecular junction formation is seen.

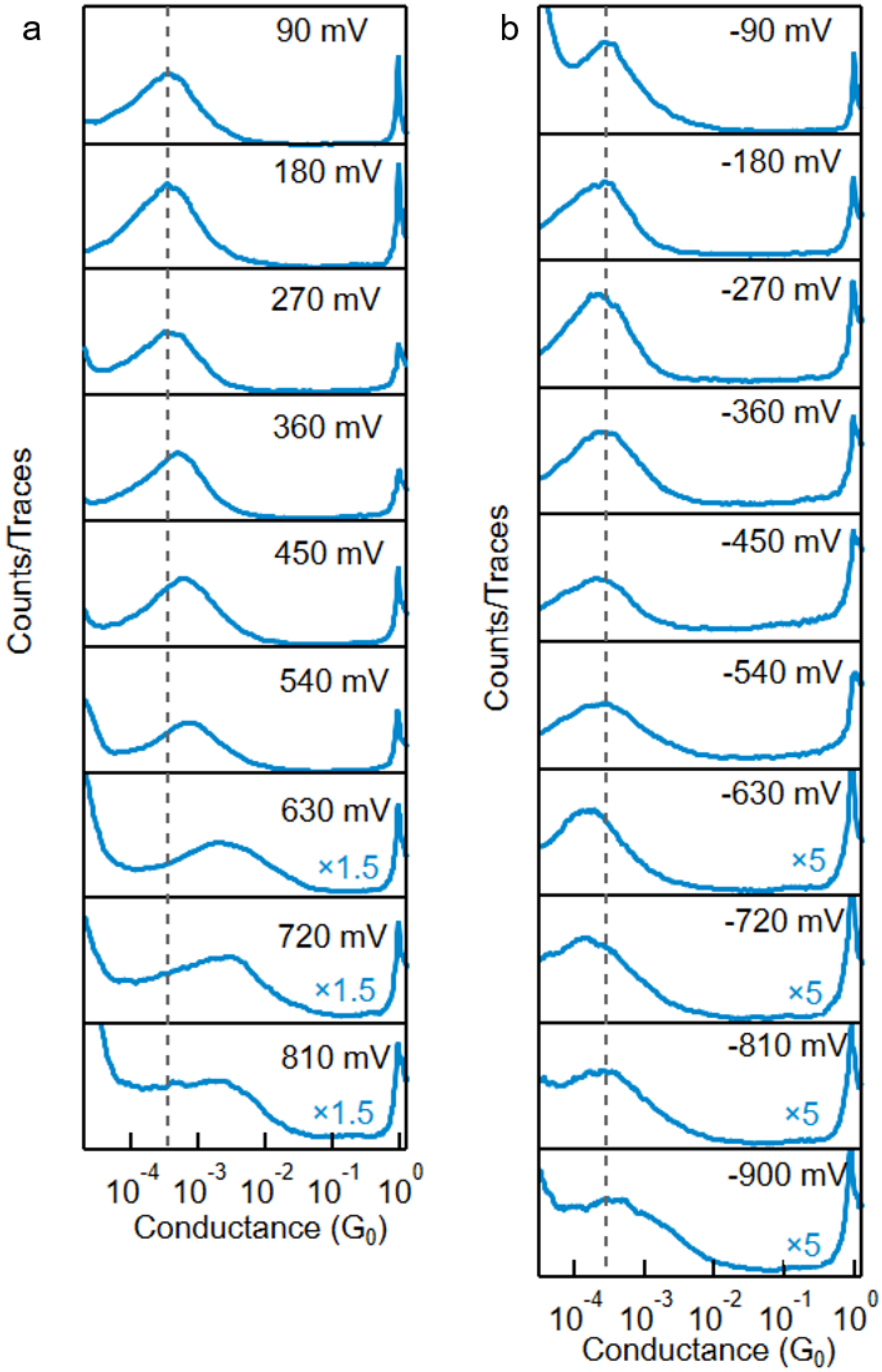

Figure S13. Logarithmically binned 1D conductance histograms for molecule **2** measured in PC under (a) positive and (b) negative bias voltages. We do not determine a single-

molecule conductance value for measurements under -810 mV and -900 mV as no clear molecular junction formation is seen in 2D histograms.

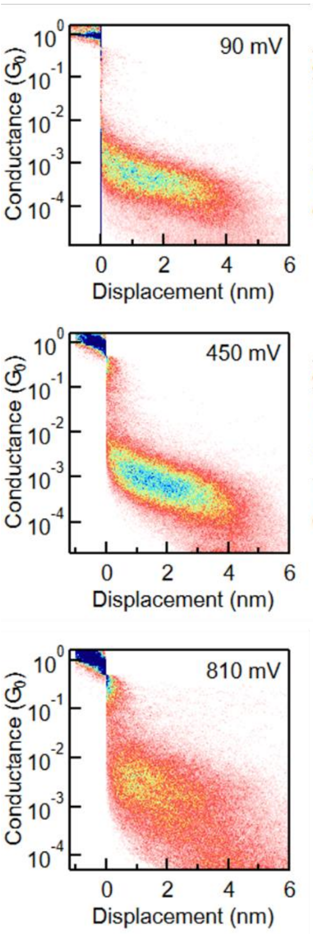

Figure S14. 2D conductance histograms of **2** measured in PC under positive tip bias voltages in the range of 90 – 810 mV.

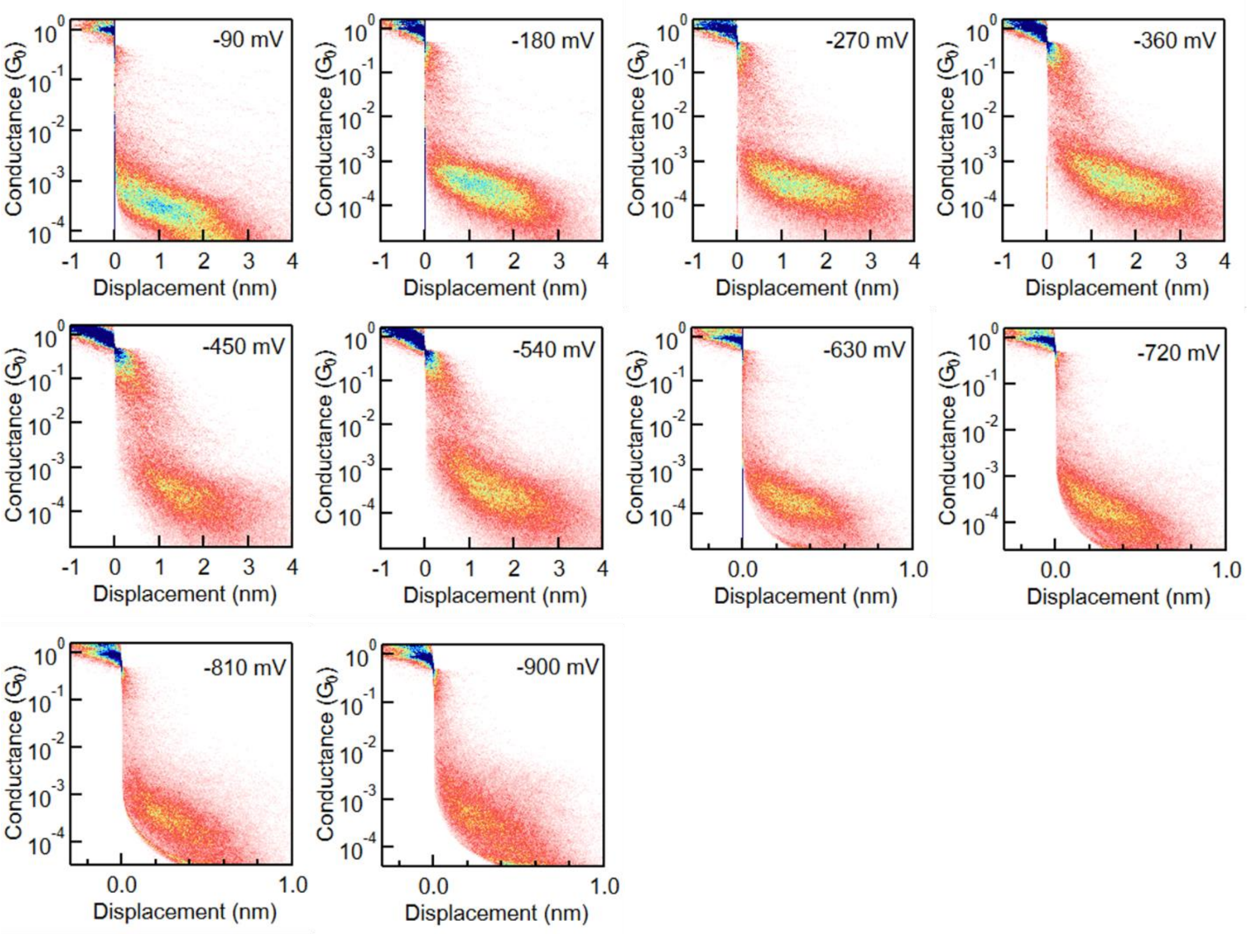

Figure S15. 2D conductance histograms of **2** measured in PC under negative tip bias voltages in the range from -90 to -900 mV. We note that the different molecular junction elongation length likely results from the use of different wax-coated tips for measurements performed above and below -540 mV. Our measurements performed with a wax-coated tip often, but not always, show an increase by a factor of 5 to 6 in molecular junction elongation length in comparison to measurements performed with a non-coated tip. We hypothesize that a change in snapback from the Au atomic contact rupture, an increased propensity for formation of Au atomic chains, or an unusual slipping of the Au tip are possible reasons for this phenomenon, and the underlying cause is being investigated. We do not determine a single-molecule conductance value for measurements under -810 mV and -900 mV as no clear molecular junction formation is seen.

III. References

1. Guo, W. Y.; Quainoo, T.; Liu, Z. F.; Li, H. X., Robust binding between secondary amines and Au electrodes. *Chemical Communications* **2024,** *60* (25), 3393-3396.

2. Hu, Z.; Fu, W. F.; Yan, L. J.; Miao, J. S.; Yu, H. T.; He, Y. W.; Goto, O.; Meng, H.; Chen, H. Z.; Huang, W., Effects of heteroatom substitution in spiro-bifluorene hole transport materials. *Chemical Science* **2016,** *7* (8), 5007-5012.
3. Li, L.; Louie, S.; Evans, A. M.; Meirzadeh, E.; Nuckolls, C.; Venkataraman, L., Topological Radical Pairs Produce Ultrahigh Conductance in Long Molecular Wires. *Journal of the American Chemical Society* **2023**, 2492-2498.
